# The effect of dry shear aligning of nanotube thin films on the photovoltaic performance of carbon nanotube–silicon solar cells

**DOI:** 10.3762/bjnano.7.141

**Published:** 2016-10-20

**Authors:** Benedikt W Stolz, Daniel D Tune, Benjamin S Flavel

**Affiliations:** 1 Institute of Nanotechnology, Karlsruhe Institute of Technology, 76021 Karlsruhe, Germany; 2 Department of Physics, Karlsruhe Institute of Technology, 76131 Karlsruhe, Germany; 3 Centre for Nanoscale Science and Technology, The Flinders University of South Australia, Adelaide 5042, Australia

**Keywords:** absorbance, carbon nanotubes, current-voltage, dry shear aligning, order parameter

## Abstract

Recent results in the field of carbon nanotube–silicon solar cells have suggested that the best performance is obtained when the nanotube film provides good coverage of the silicon surface and when the nanotubes in the film are aligned parallel to the surface. The recently developed process of dry shear aligning – in which shear force is applied to the surface of carbon nanotube thin films in the dry state, has been shown to yield nanotube films that are very flat and in which the surface nanotubes are very well aligned in the direction of shear. It is thus reasonable to expect that nanotube films subjected to dry shear aligning should outperform otherwise identical films formed by other processes. In this work, the fabrication and characterisation of carbon nanotube–silicon solar cells using such films is reported, and the photovoltaic performance of devices produced with and without dry shear aligning is compared.

## Introduction

During the last decade or so, the potential benefits of using carbon nanotubes in solar cells has been explored from both a fundamental theory point of view [1–2], as well as experimentally in a host of different device architectures, including as additives in dye solar cells [3–4], organic photovoltaics [5–6], and perovskites [7–8] and as the active light absorbing component in conjunction with acceptors such as fullerenes [9–12]. Carbon nanotube–silicon heterojunctions can also function as solar cells [13–14] and over the last few years of development the power conversion efficiency (PCE) of these devices has been steadily increasing [15–22], with the most recent high efficiency record by Wang et al. now standing at ≈17% [23]. The understanding of precisely how these junctions operate is still incomplete and although progress in this respect is ongoing [24–41], there are still many questions unanswered. What has been established is that, as one should expect, the quality of the junction plays a large role in overall performance. Since the underlying silicon base is usually a high-grade monocrystalline material, junction quality is dominated by aspects relating to the nanotube thin film. An important factor in many devices is the degree of coverage of the films on the silicon surface, itself dependent on the films’ porosity. Jung et al. [17], Li et al. [28] and Tune et al. [42] have shown that alignment of the nanotubes by solution shearing (as known as slide casting, liquid film shearing, shear force casting) from solutions of either the nanotubes dissolved natively in chlorosulphonic acid (Jung, Li), or the nanotubes dissolved as polyelectrolyte salts in polar, aprotic solvent (Tune) yields higher performing devices than their randomly oriented counterparts, and explained these observations with reference to the better surface coverage provided by such films.

Recently, the process of dry shear aligning (DSA), wherein a randomly oriented thin film of nanotubes is flattened and aligned by application of shear force in the dry state, was reported to yield similar films as those obtained by solution shearing. It was further suggested that perhaps such films may yield similar improvements in carbon nanotube–silicon solar cell performance [43]. To address this question, we report herein the results of a direct comparison between two sets of solar cells fabricated with either, a) nanotube films produced by common vacuum filtration from aqueous suspension or, b) identical films (from the same filtration membrane) that have been subjected to DSA before deposition onto the silicon surface.

## Results and Discussion

**Figure 1:**
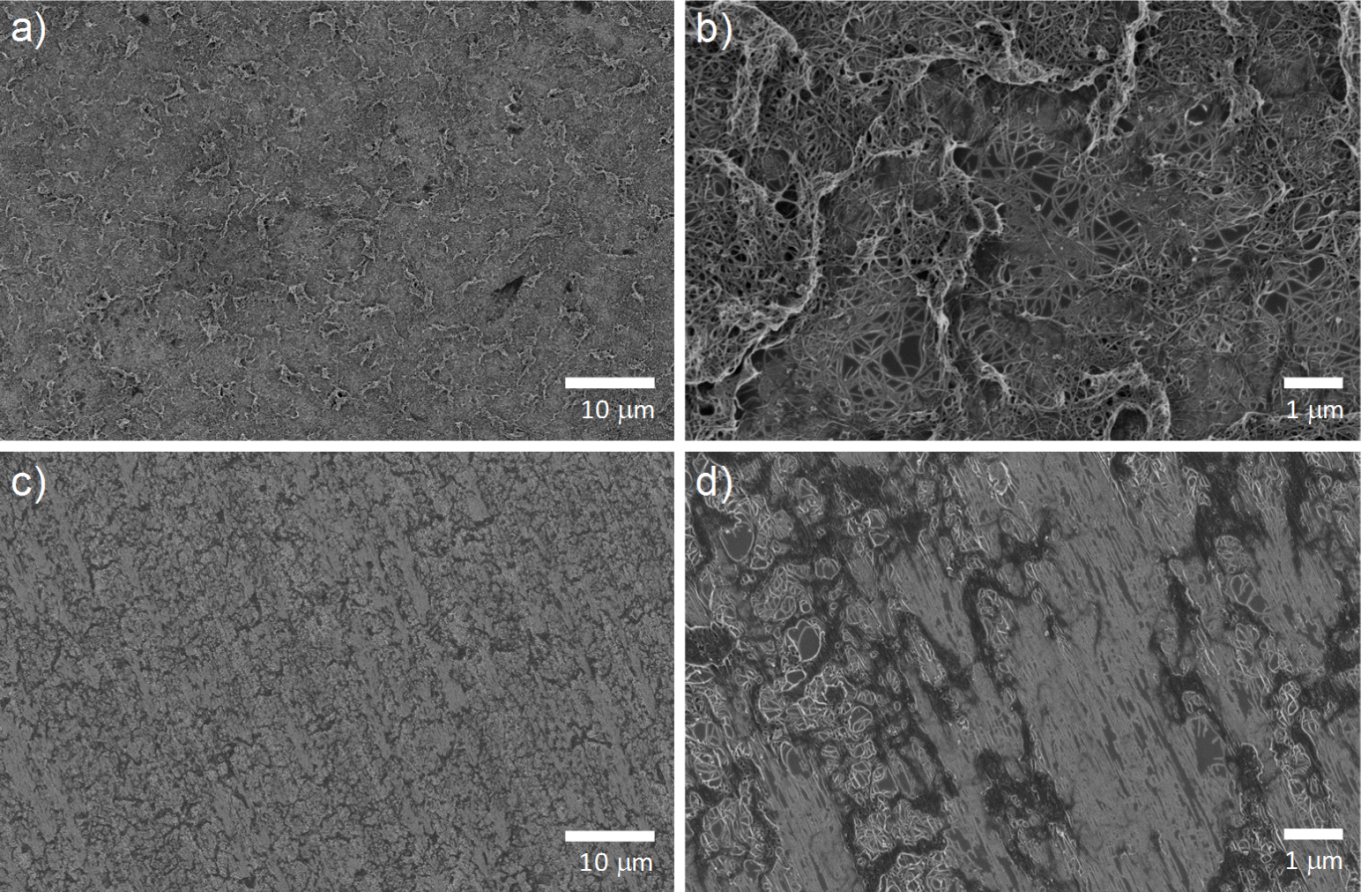
Effect of dry shear aligning (DSA) on film morphology. a) and b) show SEM images of thin films produced by vacuum filtration, before DSA, whereas c) and d) show the same films after DSA.

SEM images (Figure 1) reveals clear differences between the nanotube films as captured on the filtration membranes, and the same films after DSA. As well as the obvious alignment of the nanotubes in the direction of shear, the porosity of the film has also been reduced due to better packing of the nanotubes in their aligned configuration. The films are now also visibly flatter, with atomic force microscopy showing two orders of magnitude reductions in surface roughness (from ≈140 nm to only 3–4 nm) [43], which should lead to the desired improvements in interfacial contact between the nanotube film and the silicon substrate.

**Figure 2:**
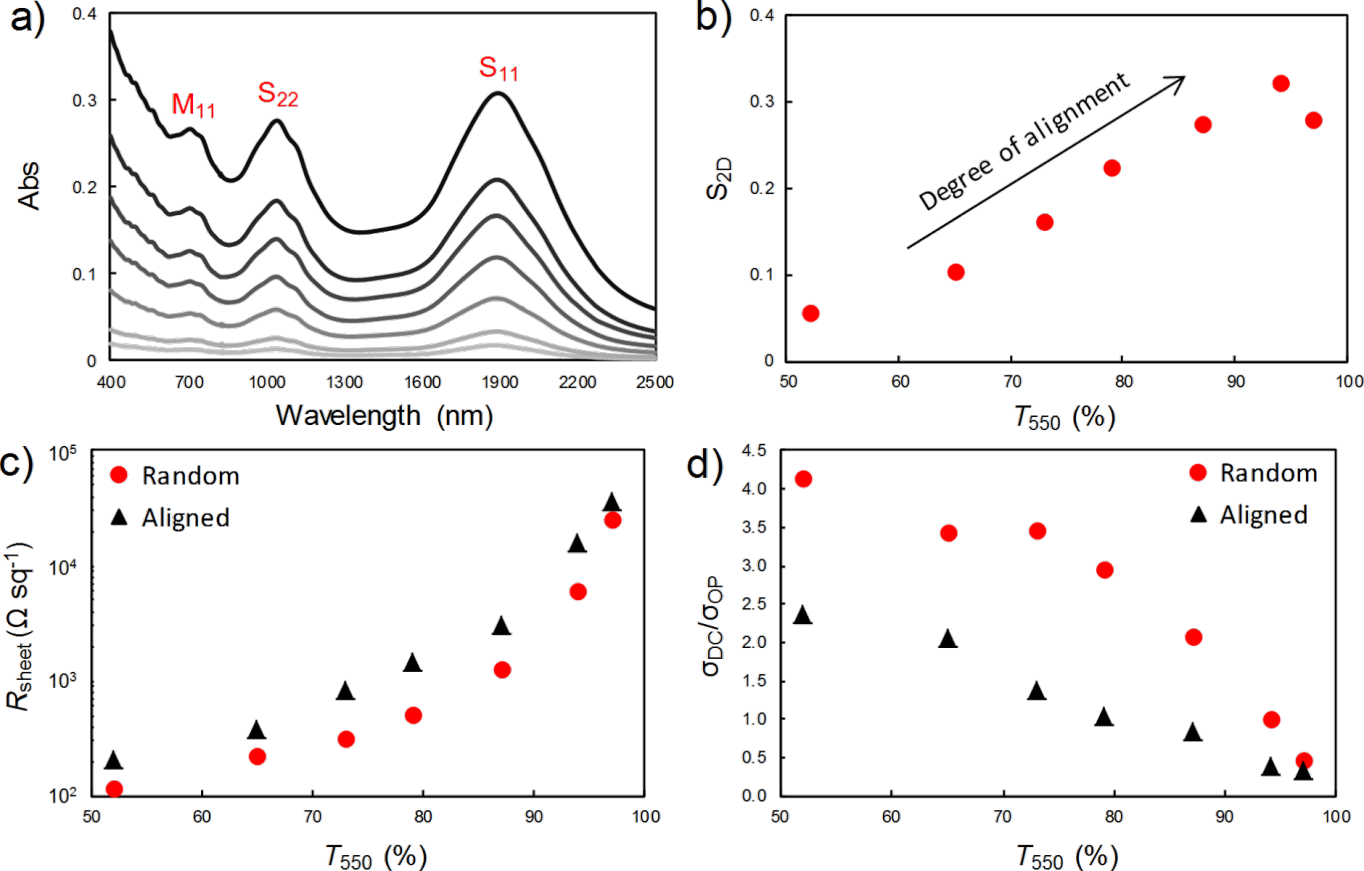
a) Optical spectra of the various thicknesses of a SWCNT film, b) variation of the degree of alignment with film thickness, c) variation of sheet resistance with film thickness for random and aligned SWCNT films and, d) variation in the ratio of DC electrical to optical conductivity with film thickness for random and aligned SWCNT films.

The optical properties of the nanotube films (Figure 2a) show the typical features of large diameter, arc discharge SWCNTs with a broad S_11_ region around 1900 nm, corresponding to a diameter distribution centred about 1.4 nm. The features on either side of the main peak are better resolved in the similarly shaped S_22_ region and indicate that there are many (n,m) species in the distribution, with the M_11_ region also displaying several components. The data is presented in Figure 2a on the familiar absorbance scale but a simple conversion yields the *T*_550_ value commonly used in the quantification of transparent films’ performance. The isotropic absorbance spectra were unchanged by DSA however, Figure 2b shows the variation in the degree of alignment of the nanotubes in the films after DSA, where the 2D order parameter is calculated from polarised optical transmittance measurements as in Equation 1 [44] and reveals that the degree of alignment increases as the film thickness decreases. This has been explained previously by considering that DSA affects mostly the nanotubes on the surface of the film, leaving the nanotubes in the ‘bulk’ of the film relatively unaffected. As the thickness decreases, the ratio of surface nanotubes to the bulk increases and the surface nanotubes thus contribute more to the overall absorbance of the film.

[1]
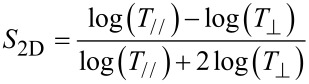


The sheet resistance of the films varies with thickness as shown in Figure 2c. Taking into account the log resistance scale, there are two main regions below and above *T*_550_ = 85–90%, corresponding to the transition between the two dominant conduction mechanisms of variable range hopping through denser films and percolation through sparse films. The measurements were taken with a linear four-point probe using a DC bias, however no anisotropy in the resistance was observed with rotation of the probe. The sheet resistance, *R*_sheet_, of the films was marginally increased after DSA, which is made clearer when considering the DC electrical to optical conductivity ratio, σ_OP_/σ_DC_ (Figure 2d), calculated as per Equation 2 [45], which takes into account the sheet resistance and the (isotropic) transmittance and where μ_0_ and ε_0_ have their usual meaning.

[2]
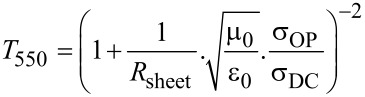


**Figure 3:**
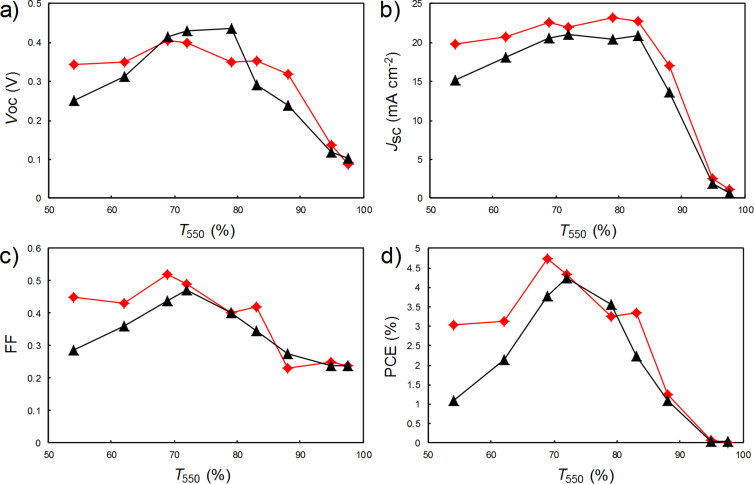
The variation of solar cell parameters with nanotube film thickness for films both before (red diamonds) and after (black triangles) DSA, a) open circuit voltage, b) short circuit current density, c) fill factor and, d) power conversion efficiency.

The as-prepared films and those aligned by DSA were deposited on silicon substrates patterned with electrodes, and the photovoltaic performance was measured, with the results summarised in Figure 3 (and with the full *J*–*V* curves shown in Supporting Information File 1, Figure S1). Whilst the DSA devices exhibited a slightly higher open circuit voltage (*V*_oc_) for films of around *T*_550_ = 70–80%, the short circuit current density (*J*_sc_) was lower for all film thicknesses, especially the thicker films, and the fill factor (FF) was also increasingly poorer tending towards thicker films. The overall result in terms of PCE is that the DSA devices performed relatively the same as the devices with as-prepared films for *T*_550_ ≥ 70%, but performed increasing worse for the thicker films, with the DSA devices of *T*_550_ = 54% generating only a third of their as-prepared film counterparts.

The reasons for the poorer performance are not immediately obvious. Although there is a clear difference in the sheet resistance, and thus DC electrical to optical conductivity ratio, it does not appear to be large enough to account for the observed difference in performance. Indeed, the two trends are somewhat different; the relative difference in sheet resistance is lowest at the two ends of the transmittance range, and is greatest in the middle of the range, whereas the relative difference in the solar cell performance (as seen most clearly in the short circuit current density, Figure 3b) is at a minimum for the thinnest films and maximum for the thickest films. It was observed that the upper side of the nanotube films on the filtration membranes became visibly more reflective after DSA and although the films were deposited with the reflective side down, it was thought that perhaps this could change the reflectance of the silicon surface differently to when an as-prepared film was deposited. However, reflectance measurements taken of the completed devices not only showed that this was not the case, but that the devices with the DSA films were, on average, 5–10% less reflective than those with the as-prepared films, across all wavelengths. Thus, when mounted on silicon, a higher photon current is penetrating through the films than the transmittance measurements suggest, which should result in a higher short circuit current density for all film thicknesses – the opposite of what is observed for the thicker films. Another possibility is that the quality of the interface between the nanotubes and silicon is inferior after DSA, though that is the opposite of what was intended. However, the diode ideality factors extracted from the dark current characteristics were similar for both the as-prepared and DSA film devices (around 1.5–2), and showed no clear trend in either case. In short, the origin of the observed performance reduction for devices fabricated with thick nanotube films that have been flattened and aligned by DSA remains undetermined, despite the fact that previous results have suggested an improvement in performance.

## Conclusion

In conclusion, a comparison was made between carbon nanotube–silicon solar cells made with either as-prepared, vacuum filtration nanotube films or the same films after flattening and aligning of the nanotubes on the contacting surface of the films using the newly developed technique of dry shear aligning. Whilst the DSA process produced films that were two orders of magnitude flatter and exhibited a 2D order parameter of up to 0.3, both of which have been previously observed to improve the photovoltaic performance of carbon nanotube–silicon junctions, in this case this was accompanied instead by a reduction in the sheet resistance and thus DC electrical to optical conductivity ratio of the films. In terms of the performance of solar cells made with the DSA films, the net result for thin films (*T*_550_ > 70%) was that there was no overall change compared to the devices with as-prepared nanotube films. However, there was a significant decrease in performance for films thicker than *T*_550_ = 70%, with the devices using the thickest DSA films (*T*_550_ = 54%) producing only a third of the photovoltaic output of the devices made with as-prepared films of the same transmittance.

## Experimental

Single walled carbon nanotubes (P2-SWNT, Carbon Solutions) were added to aqueous TritonX-100 (1% v/v, Sigma-Aldrich) at 0.1 mg mL^−1^ then bath sonicated (1 h). After centrifugation (20 000*g*, 20 min), the nanotube films were captured from the supernatant by vacuum filtration onto mixed cellulose ester (MCE) membranes (0.45 μm, HAWP, Merck Millipore) and rinsed thoroughly with copious DI water as per Wu et al. [46] and Hu et al. [47]. For the DSA preparation, a cylindrical Teflon stir bar (8 × 30 mm) was used as the aligner. The membranes were placed on a flat glass surface (nanotube side up) and held in place while the stir bar was very firmly (≈60 N) sheared across the film surface (2–3 mm s^−1^) such that approximately half of the film was thus aligned and the other half remained in the as-prepared state. The nanotube films changed in appearance from matt grey/black to become visibly smoother and more reflective, with the direction of shear faintly discernible to the eye. Small circular regions (0.08 cm^2^) of each film were then taken from both the aligned and as-prepared parts of the membranes and used in devices. The films were deposited by placing them, nanotube side down, on the solar cells such that they completely covered the active area; they were then wet with a drop of water, compressed with Teflon and baked in air (110 °C, 15 min). To remove the MCE from the films, the cooled substrates were placed in an acetone (EMSURE, Merck) bath for 30 min then transferred to two fresh acetone baths for a further 30 min each.

Carbon nanotube–silicon solar cells were fabricated as described previously [36]. Briefly, Cr/Au front electrodes were patterned onto phosphorous doped n-type silicon substrates (SSP, 525 μm thick, 1–5 Ω cm, <100>, with a 100 nm thermal oxide) by photolithography and defined circular active areas (0.08 cm^2^) in which the SiO_2_ was removed by buffered oxide etch. The films were deposited as described above and the cells were completed with GaIn eutectic back electrodes and mounted on steel support plates. Before testing, all cells were treated sequentially with 2% HF, SOCl_2_ and 2% HF again. This treatment sequence removes the thin oxide from the silicon surface as well as p-doping the nanotubes and dramatically increasing the conductivity of the nanotube films. Current–voltage data was taken from cells in the dark and under 100 mW cm^−2^ AM1.5G illumination (Class 2A, with irradiance measured by a silicon reference cell with NIST-traceable calibration).

## Supporting Information

File 1 Additional experimental information.
