# Supplementary material for: The effect of dry shear aligning of nanotube thin films on the photovoltaic performance of carbon nanotube–silicon solar cells
Source: Beilstein J Nanotechnol. 2016 Oct 20;7:1486–91. doi: 10.3762/bjnano.7.141 (PMC5082438; doi:10.3762/bjnano.7.141)
Supplement: File 1 — Additional experimental information. [file Beilstein_J_Nanotechnol-07-1486-s001.pdf]

Supporting Information

for

The effect of dry shear aligning of nanotube thin films on the photovoltaic performance of carbon nanotube–silicon solar cells

Benedikt W. Stolz[1,2], Daniel D. Tune[1,3], and Benjamin S. Flavel*[1]

Address: [1]Institute of Nanotechnology, Karlsruhe Institute of Technology, 76021 Karlsruhe, Germany, [2]Department of Physics, Karlsruhe Institute of Technology, 76131 Karlsruhe, Germany and [3]Centre for Nanoscale Science and Technology, The Flinders University of South Australia, Adelaide 5042, Australia

Email: Benjamin Flavel - benjamin.flavel@kit.edu

* Corresponding author

Additional experimental information

(a)

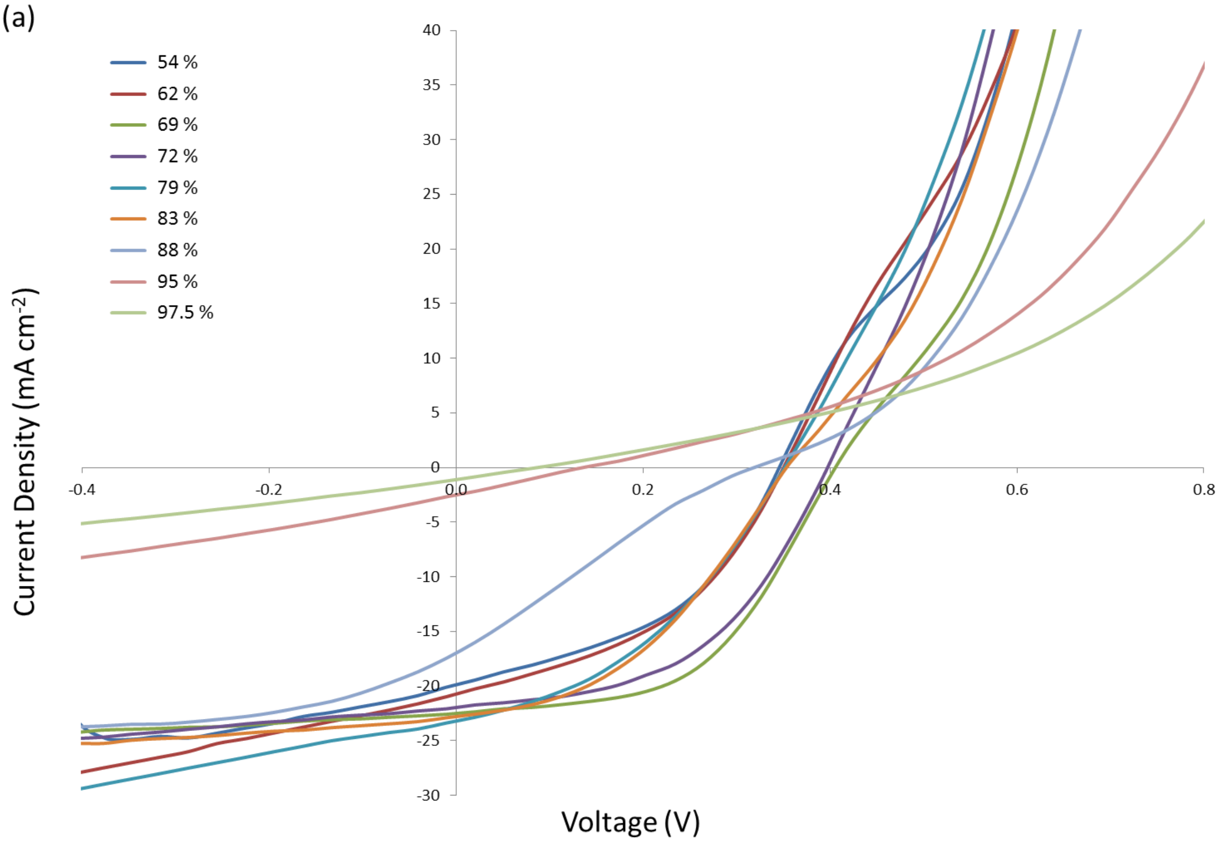

(b)

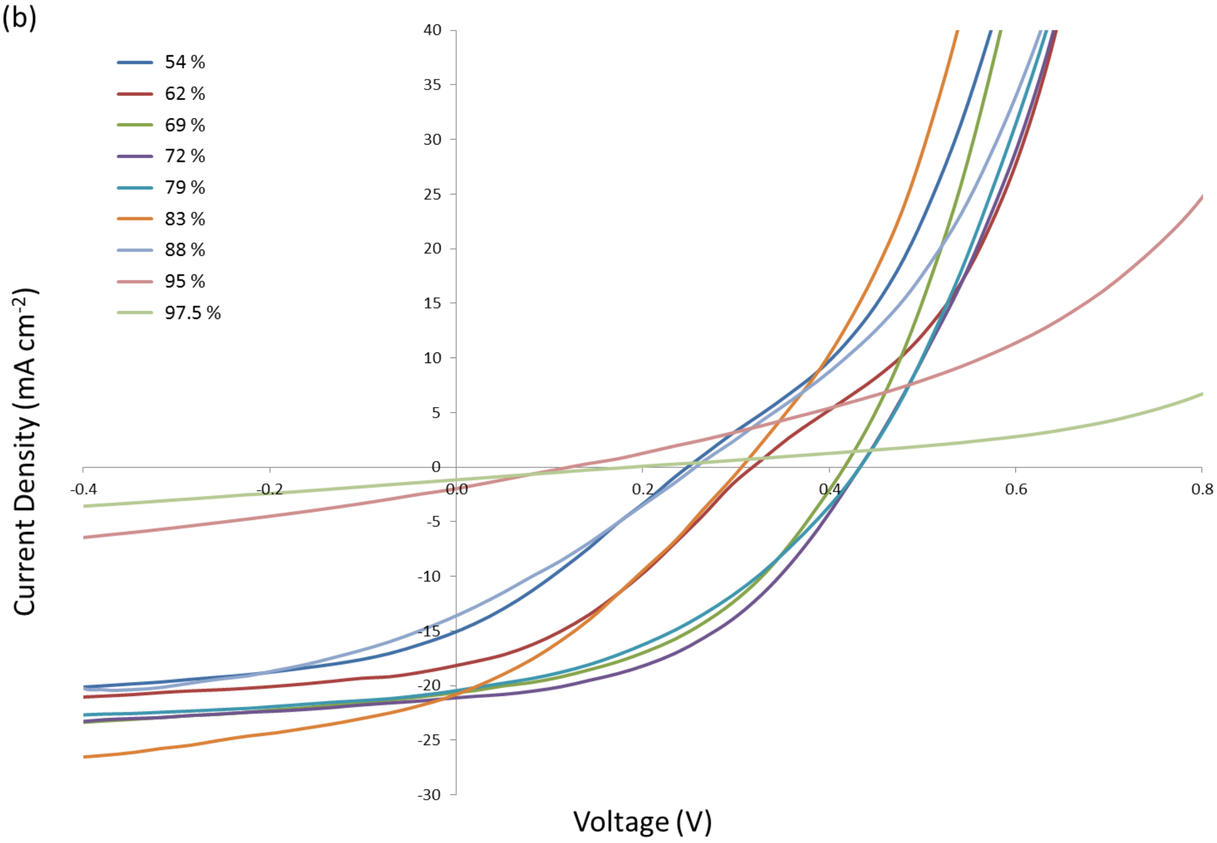

Figure S1: Variation of current density with voltage for devices made with different thicknesses (as measured by the transmittance @ 550 nm) of nanotube film using a) as prepared films or, b) dry shear aligned films. The parameters of open circuit voltage, short circuit current density, fill factor, and power conversion efficiency are summarised in Figure 3 of the main text.
